# Post-discharge outcome measurement tools in occupational therapy for people with acquired brain injury in Japan: a scoping review

**DOI:** 10.7717/peerj.20765

**Published:** 2026-03-17

**Authors:** Nozomi Oyama, Shigeharu Aoki, Tracey Williams-Macklin, Andrew Bateman

**Affiliations:** 1 School of Health and Social Care, University of Essex, Colchester, United Kingdom; 2 Department of Rehabilitation Medicine, Kanagawa Rehabilitation Hospital, Kanagawa, Japan

**Keywords:** Acquired brain injury (ABI), Outcome measurement tool, Occupational Therapy, Rehabilitation, Japan

## Abstract

**Purpose:**

Outcome measurement is fundamental to rehabilitation practice; however, the tools commonly used in occupational therapy after Acquired Brain Injury (ABI) may not adequately capture the complex needs of individuals living in the community. This scoping review synthesised the outcome measures employed in post-discharge occupational therapy in Japan and identified the core concepts they assess.

**Method:**

A comprehensive search of nine databases was conducted without restrictions on publication year or language. Search strategies were developed using relevant keywords, and four independent reviewers applied predefined inclusion and exclusion criteria to ensure methodological rigour.

**Results:**

Of the 1,188 abstracts screened, 985 articles were excluded, leaving 104 for full-text review. Ultimately, 44 studies met the eligibility criteria, yielding 32 distinct outcome measures. The Functional Independence Measure (FIM) (29.5%) was most frequently applied, followed by the Frenchay Activities Index (FAI) (9.0%), the Life Space Assessment (LSA) (7.7%), and the Barthel Index (BI) (7.7%). Categorisation of the 11 most frequently used tools according to the International Classification of Functioning, Disability, and Health (ICF) revealed a strong emphasis on mobility (26%) and self-care (18%).

**Discussion:**

These findings reflect both the influence of Japan’s ageing population on assessment priorities and a critical gap in which participation, cognition, and broader psychosocial outcomes remain underrepresented. Broader and more comprehensive assessment strategies are required to address the diverse realities of community life following ABI.

## Introduction

### Background on outcomes used in occupational therapy in Japan

Rehabilitation outcomes reflect the impact of interventions and are assessed using standardised tools to compare pre- and post-intervention status. Such tools are central to Evidence-Based Practice (EBP) (Khalifa, Magrabi & Gallego, 2019) and are essential for evaluating effectiveness at both individual and population levels (McPherson, Taylor, & Leplège, 2010). They also provide critical data for healthcare systems and policy-making.

Occupational therapists support clients holistically, addressing physical, cognitive, social, emotional, and environmental factors. Consequently, they employ a wide range of outcome measures, from Activities of Daily Living (ADL) and Instrumental Activities of Daily Living (IADL) to Quality of Life (QOL). Widely recognised tools include the Assessment of Motor and Process Skills (AMPS) (Fisher, 1995; Fisher, 1997; Fisher, 1999), Australian Therapy Outcome Measures (AusTOMs) (Enderby & John, 1997; Perry et al., 2004), Canadian Occupational Performance Measure (COPM) (Law et al., 1994; Law et al., 2005), EuroQol 5-Dimensions (EQ-5D) (Brooks R, EuroQol Group, 1996), and Health of the Nation Outcome Scales (HoNOS) (Wing, Curtis & Beevor, 1996). Although validated Japanese versions exist for some of these tools, their routine clinical use among occupational therapists in Japan remains limited.

In Japanese rehabilitation hospitals, the Functional Independence Measure (FIM) (Granger & Hamilton, 1994) is the most widely used tool. FIM scoring before and after interventions is used to determine improvement and supports the operation of specialised “convalescent rehabilitation wards”, which were established in response to the ageing population. According to the Statistics Bureau of Japan (2023), individuals aged 65 and older accounted for 29.1% of the population in 2021, the highest rate worldwide. These wards provide intensive rehabilitation for up to 180 days for individuals with specific conditions, facilitating transition to community-based care while reducing long-term care costs. The government mandates FIM use to monitor patients’ progress, making it indispensable in hospital-based occupational therapy. However, outcome measures specific to occupational therapy remain undefined both in hospital and in community-based settings. In particular, post-discharge occupational therapy lacks a set of unified and clearly defined outcome measures, reflecting the diversity of community-based interventions. This absence raises concerns about whether the tools currently used sufficiently capture the multidimensional outcomes that occupational therapy seeks to promote.

### Acquired Brain Injury and rehabilitation outcomes

For individuals with Acquired Brain Injury (ABI), outcomes are particularly complex due to diverse sequelae. ABI may result from either traumatic or non-traumatic injuries occurring after birth, such as stroke, brain tumours, encephalopathy, infections, hypoxia, and traumatic brain injury (TBI) from accidents (Goldman et al., 2022), and can lead to a wide range of consequences. These include cognitive, emotional, behavioural, and communication difficulties as well as motor and sensory impairments. While some changes are temporary, a substantial proportion result in permanent disabilities that affect functioning and reduce independence, as well as social, vocational, and recreational engagement. Consequently, many ABI survivors require ongoing rehabilitation even after discharge; therefore, an appropriate and unified outcome measure for ABI rehabilitation may be essential to support evidence-based practice.

A review by Van Heugten et al. (2020) summarised outcome measures in ABI research, with a primary focus on neuropsychological interventions. Frequently employed tools included FIM and the Hamilton Depression Rating Scale (Hamilton, 1960). However, no reviews have specifically examined post-discharge outcomes for ABI, either internationally or within Japan. Jacobs & Van Schalkwyk (2022) highlighted the importance of aligning educational and rehabilitation outcomes with patient needs in community settings. Therefore, identifying unified outcome measures for community-dwelling clients is essential not only for Japanese context but also internationally.

### Rationale for this study

The World Federation of Occupational Therapists defines occupational therapy as promoting health and wellbeing through participation in meaningful occupations that people want, need, or are expected to do (The World Federation of Occupational Therapists, 2025). Furthermore, post-ABI rehabilitation aims not only to restore lost functions (Evans, 2011) but also to maximise potential across psychological, social, occupational, and daily domains (Wilson et al., 2009). Thus, community-based occupational therapy for individuals with ABI seeks to support clients in adapting to life following cognitive or functional impairments. However, it remains unclear whether the outcome tools currently used in Japan effectively support such interventions. Identifying these tools and the concepts they assess is therefore crucial for evaluating their suitability, determining the need for new measures, and examining their alignment with occupational therapy goals.

A scoping review was selected as the most appropriate approach to map outcome measurement tools used for individuals with ABI after discharge in Japan. Scoping reviews aim to summarise existing literature and identify key concepts or evidence types (Peters et al., 2020), offering broader insights than systematic reviews, which address narrowly defined questions. While maintaining methodological rigour, scoping reviews typically omit critical appraisal or bias assessment (Peters et al., 2020). They are particularly valuable in rehabilitation science, where the scarcity of randomised controlled trials often limits the feasibility of systematic reviews (Levac, Colquhoun & O’Brien, 2010).

### Objectives

This scoping review aimed to map the range of outcome measurement tools used in occupational therapy for individuals with ABI after discharge in Japan. The review also sought to identify the key concepts assessed by these tools and to examine the extent to which these outcomes align with the core objectives of occupational therapy and ABI rehabilitation. Furthermore, the findings were used to consider whether additional or revised outcome measures may be needed. This review is intended for clinicians, researchers, and rehabilitation programme developers who require an overview of currently used outcome measures.To address these aim, the following questions guided the review:

1. What outcome measurement tools have been used in occupational therapy for individuals with ABI after discharge in Japan?
2. What concepts are assessed by these tools as rehabilitation outcomes?

## Methods

This scoping review was conducted following methodological guidance from the Joanna Briggs Institute (JBI) (JBI Scoping Review Network, 2022), Arksey & O’Malley (2005), Levac, Colquhoun & O’Brien (2010), and updated guidance (Peters et al., 2020), and was reported according to the Preferred Reporting Items for Systematic Reviews and Meta-analyses extension for Scoping Reviews (PRISMA-ScR) Protocols checklist (Tricco et al., 2018).

The review protocol was developed per JBI guidance, and a preliminary search of MEDLINE, Cochrane, and JBI Evidence Synthesis confirmed no similar reviews. The protocol was registered on the Open Science Framework on 7 September 2022 (https://doi.org/10.17605/OSF.IO/FPAB6), as protocols for scoping reviews are not currently accepted by the International Prospective Register of Systematic Reviews (PROSPERO) (Peters et al., 2020).

### Eligibility criteria

The inclusion and exclusion criteria were guided by the PCC (Population, Concept, and Context) framework (Peters et al., 2017) (Appendix 1). The “Population” included individuals of any age with ABI living in the community post-discharge; those without ABI, not receiving rehabilitation, or inpatients were excluded. The “Concept” focused on outcome measures used in occupational therapy or broader rehabilitation interventions assessing pre- and post-intervention changes in Japan. Rehabilitation interventions were included, in addition to occupational therapy, in order to avoid missing instances in which occupational therapy formed part of a multidisciplinary team intervention, given the team-based nature of rehabilitation. Tools under development or lacking Japanese validation were excluded, as were measures unrelated to rehabilitation or targeting other conditions. Tools assessing isolated functions were excluded when studies focused solely on anatomical or cognitive changes. The “Context” was community-based rehabilitation in Japan, with hospital-based tools included only if their use continued post-discharge. All study designs were eligible. No restrictions on publication year or language were applied. Details of the inclusion and exclusion criteria are summarised in Appendix 2.

### Information sources

The databases searched and justifications for their use are as follows:

1. MEDLINE Ultimate: Managed by the National Library of Medicine (NLM), this is one of the largest health science databases, covering medicine, nursing, dentistry, veterinary medicine, the health care system, and related fields.
2. PubMed Central (PMC): A full-text digital repository of biomedical and life science literature.
3. PubMed: A citation database that, unlike PMC, contains a broader range of literature primarily from MEDLINE, life science journals, and books. PubMed employs a more stringent inclusion process than PMC; while literature in PMC may also appear in PubMed, not all PubMed literature is available through PMC.
4. OTseeker: A specialised database containing resources on occupational therapy interventions.
5. CINAHL: A database covering nursing and allied health literature.
6. Cochrane Library: Comprising seven databases, including the primary database for systematic reviews. It also includes grey literature in addition to published studies.
7. Ichushi Web: The largest database for published medical literature in Japanese.
8. CiNii: A database of Japanese academic publications, consisting of three databases, one of which includes unpublished dissertations.
9. J-STAGE: A platform for scholarly publications in Japan, covering a wide range of scientific fields, including medical and health sciences. It also contains unpublished materials, such as abstract booklets of rehabilitation conferences.

Additionally, relevant stakeholders and authors were contacted when abstracts or full-text articles could not be accessed online or through an institutional library, or when such communication was required for a more comprehensive understanding of the study details.

### Search strategy

A preliminary search in MEDLINE Ultimate and Ichushi Web identified relevant keywords and Medical Subject Headings (MeSH) terms. These informed a comprehensive search strategy adapted for each database (Appendix 3). Japanese keywords corresponded to English terms to address limited English usage in Japanese publications. Grey literature, including conference proceedings, was included to capture clinical trends.

### Selection of sources of evidence

**Figure 1:**
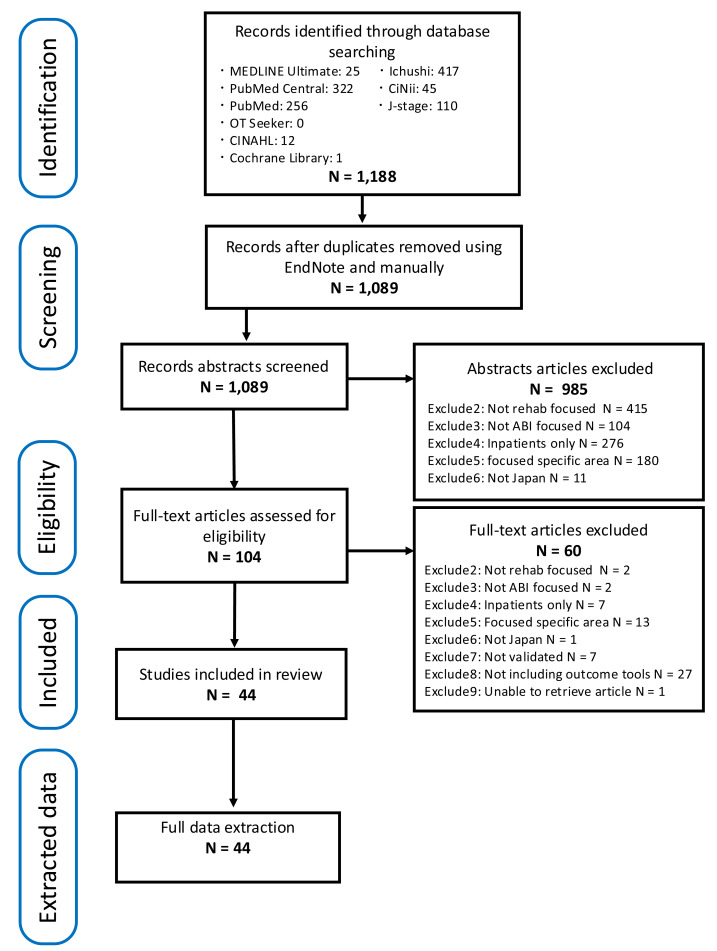
PRISMA flow diagram of study selection.

Citations were screened using titles and abstracts, with duplicates removed in EndNote. Four independent reviewers undertook the screening, and any discrepancies were resolved through discussion. The pre-resolution inter-rater agreement rates for abstract screening were 82.4% for English databases and 87.4% for Japanese databases, both exceeding the 80% threshold regarded as acceptable according to the PRISMA-ScR checklist (Tricco et al., 2018). For full-text screening, the pre-resolution inter-rater agreement rates were 88.9% for English databases and 77.2% for Japanese databases. In line with PRISMA recommendations, the study selection process was illustrated using a flow diagram (Fig. 1).

### Data charting process

English-language articles were reviewed by two native English reviewers and one Japanese reviewer, whereas Japanese-language articles were reviewed by two native Japanese reviewers. A calibration exercise was first conducted for all four reviewers to ensure a shared understanding of the inclusion criteria. Eligible citations were independently coded in Excel as “include” or “exclude”, with reasons for exclusion recorded. Minor clarifications to the criteria were agreed upon by consensus; however, individual screening results were not shared in order to minimise selection bias. Once all reviewers had completed screenings, the datasets were consolidated using a separate Excel spreadsheet, enabling online discussions and the calculation of inter-rater agreement rates.

### Data items

Data extraction followed a tool aligned with JBI guidance (JBI Scoping Review Network, 2022) and the PRISMA-ScR (Tricco et al., 2018). The tool included specific details and results related to the PCC framework of this scoping review. The following information was collected:

**Authors and year**: Lead author and year of publication.

**Name of instruments**: Instruments used in the article as outcome measures. Validation of the Japanese version tools was confirmed during full-text screening, by referring to other relevant articles that reported their validation.

**Outcome concept assessed**: The main concept assessed by the outcome tools, clarifying which aspects were emphasised in the literature.

**Number of items**: The number of items, subscales, or questions included in the outcome tools, with examples or concise descriptions where available. If articles did not provide sufficient detail, relevant literature was consulted.

**Mode of administration**: The individual(s) responsible for administering the instruments (*e.g.*, self-report, interview, or observation).

**Study design**: The study designs employed. When not explicitly stated, it was inferred from the content.

**Target population**: The population described, including type of brain injury, type of rehabilitation received in the community, and/or their living arrangement after discharge.

**Sample size**: The number of participants included.

**Age range and mean**: The age range and mean age of participants.

**Language**: The primary language used in the article.

### Synthesis of results

Findings were summarised by the principal reviewer and confirmed with the team. The number and types of outcome measures, their concepts, and trends over time were presented graphically and narratively. Frequently used instruments were classified into the domains of International Classification of Functioning, Disability and Health (ICF) (WHO, 2001), following previous reviews by Tate, Godbee & Sigmundsdottir (2013), which utilised the ICF classification to classify assessment tools for individuals with TBI. This approach was similarly applied in a systematic review conducted by Hoffman et al. (2014), which examined health outcome measures for rehabilitation in patients with major trauma.

## Results

### Selection of sources of evidence

A total of 1,089 citations were identified after duplicate removal. Details of the search process are shown in Fig. 1. The majority of records originated from Ichushi Web, followed by PubMed Central. After title and abstract screening, 985 articles were excluded (515 from English and 470 from Japanese databases, including 72 grey literature sources), leaving 104 for full-text article assessment. Detailed reasons for exclusion and their respective counts are provided in Fig. 1. At this stage, the reasons for exclusion were categorised into five groups, Exclude 2: Not rehabilitation (rehab) focused, Exclude 3: Not ABI-focused, Exclude 4: Inpatients only, Exclude 5: Focused on a specific area, and Exclude 6: Not related to Japan (Exclude 1, referring to duplicate, is not shown). Subsequently, during full-text screening, a further 60 articles were excluded. The reasons for exclusion, as shown in Fig. 1, included two new categories; Exclude 7: Not validated, and Exclude 8: Not including outcome tools. In addition, one grey literature source was excluded because the full text could not be obtained (Exclude 9). Finally, 44 full text articles were deemed eligible for data extraction.

### Characteristics and results of individual sources of evidence

The characteristics of the 44 included studies are summarised as follows: English-language studies are shown in Table 1 (*n* = 5), Japanese-language studies in Table 2 (*n* = 19), and Japanese grey literature in Appendix 4 (*n* = 20). The earliest study among the included articles was published in 2005. Nearly half of the studies (43.1%) employed multiple outcome measures. While studies focusing solely on specific functions (*e.g.*, limb movement, language, cognitive function) were generally excluded, some function-specific instruments were included when used within a broader rehabilitation framework. For example, in a study by Kurokawa & Ohori (2021), Timed Up and Go Test (TUG) (Podsiadlo & Richardson, 1991) and Berg Balance Scale/Functional Balance Scale (BBS/FBS) (Berg et al., 1989) were extracted alongside the FIM to examine changes in overall functioning and quality of life. A complete list of outcome instruments is presented in Appendix 5.

**Table 1:** Source of evidence (English literature).

**Authors and year**	**Name of instrument used**	**Outcome domain assessed**	**The number of items**	**Mode of administration**	**Study design**	**Target population**	**Sample size**	**Age range and mean**	**Language**
Ashizawa et al. (2022)	The SEPA scale	Self-efficacy for physical activities	4 items related to physical activities (walking, stair-climbing, weightlifting and push-ups) *Only a walking item was used in this article	Self-report	Randomised controlled study	Patients with minor ischaemic stroke hospitalised and discharged from an acute hospital	86 (interventions = 43, controls = 43)	71.6 (±8.0)	English
	GDS15	Mood	15 items to assess depressive symptoms						
	PSQI	Sleep state	9 questions to assess sleep state						
Hashimoto et al. (2006)	FIM/FAM	ADL, motor function, cognitive function, communication, IADL	30 subscales (eating, grooming, bathing, dressing upper body, dressing, lower body, toileting, swallowing, bladder management, bowel management, transfers-bed/chair/wheelchair, transfers-toilet, transfers-bath/shower, transfers-car, walk/wheelchair, stairs, community access, comprehension, expression, reading, writing, speech intelligibility, social interaction, emotional status, adjustment to limitations, employability, problem solving, memory, orientation, attention, safety judgement)	Observation	Non-randomised controlled study	Patients with ABI joined the comprehensive day treatment programme after discharge outpatients with TBI	37 (participants = 25, controls = 12)	participants = 26.6 (±9.7) controls = 28.7(±10.9)	English
	CIQ	IADL (home, social and productive activities)	15 items (5 for home integration, 6 for social integration and 4 for productive activities)	Self-report or interview					
Kashihara et al. (2011)	FIM	ADL, motor function, cognitive function, communication	18 subscales (eating, grooming, bathing, dressing upper body, dressing, lower body, toileting, bladder management, bowel management, transfers-bed/chair/wheelchair, transfers-toilet, transfers-bath/shower, walk/wheelchair, stairs, comprehension, expression, social interaction, problem solving, memory)	Observation	Retrospective observational study	Severe stroke survivors discharged from the hospital	51	70.5 (±10.1)	English
	BI	ADL, motor function	10 subscales (feeding, bathing, grooming, dressing, bowels, bladder, toilet use, transfers, mobility, stairs)						
Mutai et al. (2013)	mRS	The degree of disability or dependence	A single scale	Self-report or report from family	Retrospective observational study	Stroke survivors discharged from the convalescent rehabilitation ward to their home	252	72.4 (±10.8)	English
	GDS15	Mood	15 items to assess depressive symptoms						
	FAI	IADL	15 subscales including 36 items (preparing main meals, washing up after meals, washing clothes, light housework, heavy housework, local shopping, social occasions, walking outside for>15 min, actively pursuing hobby, driving car/going on bus, travel outing/car ride, gardening, household maintenance, reading books, gainful work)						
Mutai et al. (2016)	FIM	ADL, motor function, cognitive function, communication	18 subscales (eating, grooming, bathing, dressing upper body, dressing, lower body, toileting, bladder management, bowel management, transfers-bed/chair/wheelchair, transfers-toilet, transfers-bath/shower, walk/wheelchair, stairs, comprehension, expression, social interaction, problem solving, memory)	Interview for participants or their family	Retrospective observational study	Stroke survivors discharged from the convalescent rehabilitation ward to their home	27	69.4 (±9.9)	English
	SF-36	Health related QOL	8 subscales (physical functioning, role physical, bodily pain, general health, vitality, social functioning, role emotional, mental health)	Interview for participants or their family					
	FAI	IADL	15 subscales including 36 items (preparing main meals, washing up after meals, washing clothes, light housework, heavy housework, local shopping, social occasions, walking outside for>15 min, actively pursuing hobby, driving car/going on bus, travel outing/car ride, gardening, household maintenance, reading books, gainful work)	Interview for participants or their family					
	HDRS	Mood	17 or 21 questions related to signs of depression	interview for participants or their family					

**Table 2:** Source of evidence (Japanese literature).

**Authors and year**	**Name of instrument used**	**Outcome domain assessed**	**The number of items**	**Mode of administration**	**Study design**	**Target population**	**Sample size**	**Age range and mean**	**Language**
Kanamoto et al. (2022)	LSA	A range, frequency and independence level of activities	15 questions regarding range, frequency and independence level in rooms of home beside the room for sleeping, an area outside home, places in neighbourhood, places outside neighbourhood, places outside a town	Self-report or interview	Retrospective observational study	Stroke survivors discharged from the convalescent rehabilitation ward to their home	23	62.2 (±11.6)	Japanese
	FES	Fear of falling	10 items that describe different activities of daily living (take a bath or shower, reach into cabinets or closets, walk around the house, prepare meals not requiring carrying heavy or hot objects, get in and out of bed, answer the door or telephone, get in and out of chair, getting dressed and undressed, cleaning the house, and going to the shop) *a modified version by Japanese Physical Therapy Association was used in this article	Self-report or interview					
	LSNS-6	Social engagement	6 items to assess the degree of active and intimate social networks with family and friends	Self-report or interview					
Kurokawa & Ohori (2021)	FIM	ADL, motor function, cognitive function, communication	18 subscales (eating, grooming, bathing, dressing upper body, dressing, lower body, toileting, bladder management, bowel management, transfers-bed/chair/wheelchair, transfers-toilet, transfers-bath/shower, walk/wheelchair, stairs, comprehension, expression, social interaction, problem solving, memory)	Observation	Case report	A stroke survivor received a day care rehabilitation after discharge from the convalescent rehabilitation ward	1	70s	Japanese
	TUG	Motor function	No item (measured time for the task)						
	BBS/FBS	Motor function	14 items (sitting to standing, standing unsupported, sitting unsupported, standing to sitting, transfers, standing with eyes closed, standing with feet together, reaching forward with outstretched arm, retrieving object from floor, turning to look behind, turning 360 degrees, placing alternate foot on stool, standing with one foot in front, standing on one foot)						
Kobayashi & Kobayashi (2021)	FAI	IADL	15 subscales (preparing main meals, washing up after meals, washing clothes, light housework, heavy housework, local shopping, social occasions, walking outside for >15 min, actively pursuing hobby, driving a car/going on a bus, travel outing/car ride, gardening, household maintenance, reading books, gainful work) *Only first 6 items were used in this article	Self-report or interview	Prospective observational study	Stroke survivors discharged from the convalescent rehabilitation ward to their home	46	73.1 (±9.0)	Japanese
Yamada (2020)	FIM	ADL, motor function, cognitive function, communication	18 subscales (eating, grooming, bathing, dressing upper body, dressing, lower body, toileting, bladder management, bowel management, transfers-bed/chair/wheelchair, transfers-toilet, transfers-bath/shower, walk/wheelchair, stairs, comprehension, expression, social interaction, problem solving, memory)	Observation	Case report	Stroke outpatients discharged from the convalescent rehabilitation ward to her home	1	70s	Japanese
	BI	ADL, motor function	10 subscales (feeding, bathing, grooming, dressing, bowels, bladder, toilet use, transfers, mobility, stairs)						
Kibayashi, Nakamoto & Tsuda (2020)	FIM	ADL, motor function, cognitive function, communication	18 subscales (eating, grooming, bathing, dressing upper body, dressing, lower body, toileting, bladder management, bowel management, transfers-bed/chair/wheelchair, transfers-toilet, transfers-bath/shower, walk/wheelchair, stairs, comprehension, expression, social interaction, problem solving, memory)	Observation	Case report	Stroke survivors discharged from the convalescent rehabilitation ward to their home	1	68	Japanese
Kobayashi & Kobayashi (2019)	FAI	IADL	15 subscales (preparing main meals, washing up after meals, washing clothes, light housework, heavy housework, local shopping, social occasions, walking outside for >15 min, actively pursuing hobby, driving a car/going on a bus, travel outing/car ride, gardening, household maintenance, reading books, gainful work) *Only first 6 items were used in this article	Self-report or interview	Retrospective cohort study	Stroke survivors discharged from the convalescent rehabilitation ward to their home	128	69.9 (±11.7)	Japanese
Takano & Teraoka (2019)	CAOD Scale	Occupational dysfunction	16 questions related to occupational dysfunctions (6 items for occupational marginalisation, 4 items for occupational imbalance, 3 items for occupational deprivation, 3 items for occupational alienation)	Self-report	Case report	A stroke survivor received a home visit rehabilitation	1	60 s	Japanese
Koshinaka et al. (2019)	FIM	ADL, motor function, cognitive function, communication	18 subscales (eating, grooming, bathing, dressing upper body, dressing, lower body, toileting, bladder management, bowel management, transfers-bed/chair/wheelchair, transfers-toilet, transfers-bath/shower, walk/wheelchair, stairs, comprehension, expression, social interaction, problem solving, memory)	Observation	Case report	Stroke survivors discharged from the convalescent rehabilitation ward to their home	1	80 s	Japanese
Hisahori et al. (2019)	Flow-FIM	ADL, motor function, cognitive function, communication	18 subscales (eating, grooming, bathing, dressing upper body, dressing, lower body, toileting, bladder management, bowel management, transfers-bed/chair/wheelchair, transfers-toilet, transfers-bath/shower, walk/wheelchair, stairs, comprehension, expression, social interaction, problem solving, memory) *Only motor items were used in this article	Self-report or interview by family or significant others	Retrospective observational study	People with ABI discharged from the convalescent rehabilitation ward to their home	37	68.03 (±14.18)	Japanese
	LSA	A range, frequency and independence level of activities	15 questions regarding range, frequency and independence level in rooms of home beside the room for sleeping, an area outside home, places in neighbourhood, places outside neighbourhood, places outside a town						
	FAI	IADL	15 subscales (preparing main meals, washing up after meals, washing clothes, light housework, heavy housework, local shopping, social occasions, walking outside for >15 min, actively pursuing hobby, driving a car/going on a bus, travel outing/car ride, gardening, household maintenance, reading books, gainful work)						
Onoda & Takeda (2018)	LSA	A range, frequency and independence level of activities	15 questions regarding range, frequency and independence level in rooms of home beside the room for sleeping, an area outside home, places in neighbourhood, places outside neighbourhood, places outside a town	Self-report	Retrospective observational study	People with ABI discharged from the acute ward or the convalescent rehabilitation ward to their home	74	68.1 (±12.6)	Japanese
Yamamoto et al. (2017)	FIM	ADL, motor function, cognitive function, communication	18 subscales (eating, grooming, bathing, dressing upper body, dressing, lower body, toileting, bladder management, bowel management, transfers-bed/chair/wheelchair, transfers-toilet, transfers-bath/shower, walk/wheelchair, stairs, comprehension, expression, social interaction, problem solving, memory)	Interview for patients and their family	Retrospective observational study	Stroke survivors discharged from the convalescent rehabilitation ward to their home	7	73 (±8.4)	Japanese
Tsuchiyama et al. (2016)	LSA	A range, frequency and independence level of activities	15 questions regarding range, frequency and independence level in rooms of home beside the room for sleeping, an area outside home, places in neighbourhood, places outside neighbourhood, places outside a town	Self-report	Retrospective observational study	Stroke survivors discharged from the convalescent rehabilitation ward to their home	18	65.7 (±11.8)	Japanese
Takashina et al. (2016)	LSA	A range, frequency and independence level of activities	15 questions regarding range, frequency and independence level in rooms of home beside the room for sleeping, an area outside home, places in neighbourhood, places outside neighbourhood, places outside a town	Self-report	Case report	Stroke survivors discharged from the convalescent rehabilitation ward to their home	1	50 s	Japanese
Kato, Fukuzawa & Shibao (2014)	FIM	ADL, motor function, cognitive function, communication	18 subscales (eating, grooming, bathing, dressing upper body, dressing, lower body, toileting, bladder management, bowel management, transfers-bed/chair/wheelchair, transfers-toilet, transfers-bath/shower, walk/wheelchair, stairs, comprehension, expression, social interaction, problem solving, memory)	Observation	Retrospective observational study	Stroke survivors being admitted into a support facility for people with disabilities	35	51.0 (±9.0)	Japanese
	10MWT	Motor function	No item (walking speed)						
	6MWT	Motor function	No item (walking distance)						
	CS-30	Motor function	No item (measured time for the task)						
	TUG	Motor function	No item (measured number times for the task)						
Murase & Watanabe (2014)	FIM	ADL, motor function, cognitive function, communication	18 subscales (eating, grooming, bathing, dressing upper body, dressing, lower body, toileting, bladder management, bowel management, transfers-bed/chair/wheelchair, transfers-toilet, transfers-bath/shower, walk/wheelchair, stairs, comprehension, expression, social interaction, problem solving, memory)	Observation	Case report	An outpatient with CVA	1	50 s	Japanese
Kanou et al. (2012)	FIM	ADL, motor function, cognitive function, communication	18 subscales (eating, grooming, bathing, dressing upper body, dressing, lower body, toileting, bladder management, bowel management, transfers-bed/chair/wheelchair, transfers-toilet, transfers-bath/shower, walk/wheelchair, stairs, comprehension, expression, social interaction, problem solving, memory)	Observation	Retrospective observational study	Outpatients with CVA discharged from the convalescent rehabilitation ward to their home	9	64.2	Japanese
Sakuma & Sawada (2011)	FIM	ADL, motor function, cognitive function, communication	18 subscales (eating, grooming, bathing, dressing upper body, dressing, lower body, toileting, bladder management, bowel management, transfers-bed/chair/wheelchair, transfers-toilet, transfers-bath/shower, walk/wheelchair, stairs, comprehension, expression, social interaction, problem solving, memory)	Observation	Single case experimental design	Stroke survivors discharged from the convalescent rehabilitation ward to their home	1	60 s	Japanese
	POMS	Mood	65 questions to assess anger, confusion, depression, fatigue, tension, vigor, and friendliness	Self-report					
Arao et al. (2009)	FIM	ADL, motor function, cognitive function, communication	18 subscales (eating, grooming, bathing, dressing upper body, dressing, lower body, toileting, bladder management, bowel management, transfers-bed/chair/wheelchair, transfers-toilet, transfers-bath/shower, walk/wheelchair, stairs, comprehension, expression, social interaction, problem solving, memory) *a questionnaire version (Ota et al., 1997) was used for assessment after discharge	Questionnaire for carers	Retrospective observational study	Stroke survivors discharged from the convalescent rehabilitation ward to their home	40	66.4 (±10.7)	Japanese
Yoshino, Sasaki & Usuda (2008)	FIM	ADL, motor function, cognitive function, communication	18 subscales (eating, grooming, bathing, dressing upper body, dressing, lower body, toileting, bladder management, bowel management, transfers-bed/chair/wheelchair, transfers-toilet, transfers-bath/shower, walk/wheelchair, stairs, comprehension, expression, social interaction, problem solving, memory)	Observation	Retrospective observational study	Stroke survivors discharged from the convalescent rehabilitation ward to their home	117	70.0 (±13.8)	Japanese

Retrospective designs were most frequent (*n* = 19, 43.2%), primarily comparing hospitalisation data with post-discharge outcomes. Almost half of the studies (45.5%) involved stroke survivors who were within one year post-onset and had been discharged from convalescent rehabilitation wards to home. No systematic reviews were identified, and only one randomised controlled trial (Ashizawa et al., 2022) investigated an intervention to reduce sedentary behaviour among 86 participants. Most studies (75.0%) targeted individuals aged 50 years or older, while only a small number of studies focused on younger populations under 40 years of age.

### Synthesis of results

**Figure 2:**
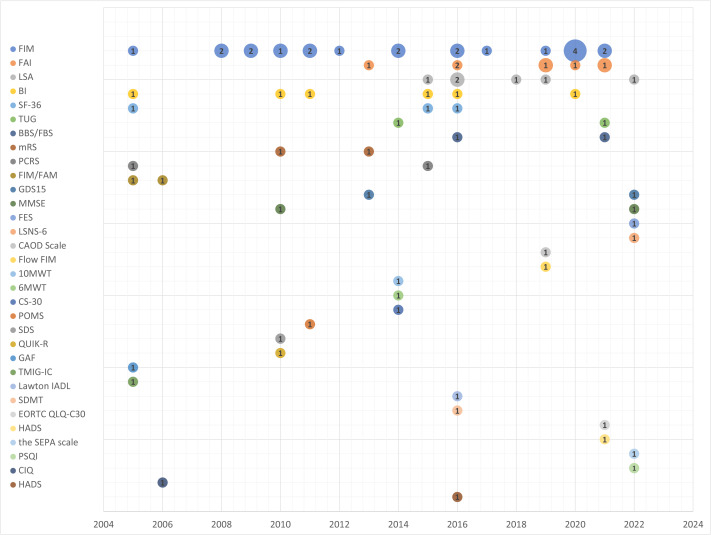
Publication year and outcome measures identified in this review. The FIM has been consistently used in Japan since the 2000s. Similarly, the LSA and the FAI began to be used mainly from around 2015. These years correspond to the timing of their adoption through governmental and public initiatives. In contrast, no clear temporal trends were observed for the other outcome measures.

Across 44 studies, 32 distinct outcome instruments were identified (Appendix 5). The most frequently used tools were the FIM (21 studies, 29.5%), followed by the Frenchay Activities Index (FAI) (Holbrook & Skilbeck, 1983) (seven studies, 9.0%) and both the Life Space Assessment (LSA) (Baker, Bonder & Allman, 2003) and Barthel Index (BI) (Mahoney & Barthel, 1965) (six studies each, 7.7%). Six outcome measures were used twice, and 21 were used once. Figure 2 illustrates the distribution of instruments by publication year, with bubble sizes representing the numbers of citations. It shows consistent use of FIM since 2005 and increasing use of the LSA, FAI, and Medical Outcomes Study Short Form-36 Health Survey (SF-36) (Stewart & Ware, 1992; Ware & Sherbourne, 1992; McHorney, Ware & Kosinski, 1993; Mchorney et al., 1994; Ware Jr, 1996) in more recent years.

**Figure 3:**
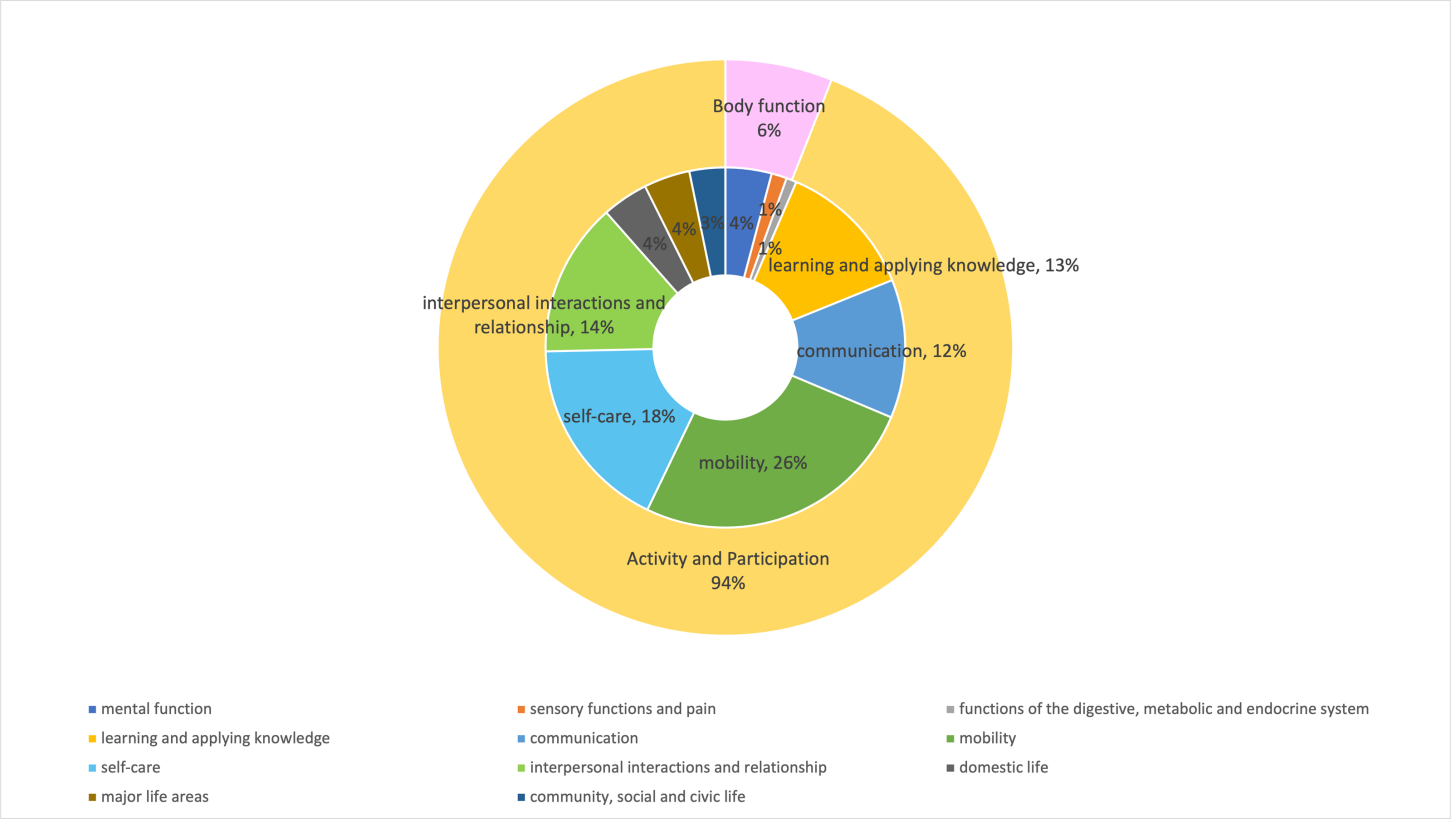
Percentage of ICF domains covered by the top 11 outcome instruments identified in this review. Activity and Participation emerged as the most prominent first-level of ICF domains. Within this domain, mobility and self-care were the most frequently assessed concepts in post-discharge occupational therapy for individuals with ABI in Japan.

The 11 most frequently cited tools (used in more than two studies) were categorised according to the ICF (Appendix 6). Most instruments addressed multiple ICF domains, with the Functional Independence Measure/Functional Assessment Measure (FIM/FAM) (Hall et al., 1992) encompassing the broadest range. In contrast, tools such as LSA, TUG, BBS/FBS, and GDS-15 were more domain-specific. Figure 3 presents the percentage distribution of ICF domains represented by the identified outcome tools, based on their frequency of use (Appendix 5) and their classification (Appendix 6). At the first-level category, 94% of the identified instruments assessed the Activity and Participation domain, 6% focused on Body Functions, and none addressed Body Structures or Environmental Factors. At the second ICF level, Mobility (d4) was the most frequently represented domain (26%), followed by Self-care (d5) (18%). Other domains, including Learning and applying knowledge (d1), Communication (d3), and Interpersonal interactions and relationships (d7), each accounted for 12–14%.

## Discussion

### Summary of evidence

This scoping review summarised outcome measures used in post-discharge occupational therapy for individuals with ABI in Japan and clarifies the concepts these tools assess.

#### Most frequently used instruments

The FIM emerged as the most frequently used comprehensive outcome measure, consistent with its global adoption since its first edition (Keith et al., 1987). Developed in 1984 by the American Academy of Physical Medicine and Rehabilitation and the American Congress of Rehabilitation Medicine, the FIM has demonstrated robust validity, reliability, and cross-cultural applicability.

In Japan, the FIM has been widely used since 2001 and is mandated in rehabilitation wards by the government to address the needs of an aging society. Although this review focused on the post-discharge context, many studies used retrospective hospital data in which FIM scores were routinely collected to compare patients’ conditions during hospitalisation and after discharge. FIM data are readily accessible because reporting is mandatory, which likely contributed to its high citation frequency in this review. Therefore, its prevalence may not reflect actual use in community-based clinical practice.

Several factors explain the government-led adoption and widespread use of the FIM in Japan, including its conceptual clarity and alignment with societal priorities. Although the FIM primarily measures activity levels, it more accurately reflects caregiving burden, determined by the degree of assistance or specialised equipment required. This emphasis is particularly relevant in Japan, which has the world’s highest proportion of older adults. The growing demand for efficient caregiving strategies has made the FIM indispensable. Moreover, because the concept of caregiving burden is easily understood, a wide range of healthcare professions can apply and score the FIM without specialised rehabilitation knowledge. This accessibility likely contributes to its popularity, especially in settings requiring interdisciplinary collaboration.

In contrast, the FAI, the second most frequently used tool in this review, was mainly applied in community settings and case reports rather than hospital-based retrospective studies, where the FIM predominated. The FAI, originally developed by Holbrook & Skilbeck (1983), provides a broader assessment of everyday activities, with its validity and reliability well established (Wade, Leigh-Smith & Hewer, 1985; Schuling et al., 1993). Although the Japanese version was created by Shiratsuchi, Saeki & Hachisuka (1999), its use has expanded in recent years, possibly due to its inclusion in CHASE (Care, Health, Status, and Events), a governmental database launched in 2020. CHASE collects caregiving data to promote “scientific caregiving” based on evidence-based practice. It succeeded the VISIT (monitoring & eValuation for rehabIlitation ServIces for long-Term care) system, which began in 2017 to monitor rehabilitation and long-term care. CHASE later broadened its scope to include living conditions and caregiving activities, adding the FAI as an assessment item. In 2021, CHASE and VISIT were integrated into a unified project called LIFE (Long-term care Information system For Evidence) (Ministry of Health, Labour and Welfare of Japan, 2022).

The BI, the third most frequently used tool, is also required for reporting in LIFE. Developed in 1965, it is an internationally recognised measure of ADL (Mahoney & Barthel, 1965). Its strength lies in simplicity: ten items scored on a 2 to 4 scale in increments of five points. Similar to the FIM, the BI score is linked to reimbursement, as maintenance or improvement can qualify for additional government subsidies.

Likewise, the LSA has been used in Japan since 2015, with its popularity influenced by public initiatives. Developed by Baker, Bonder, and Allman in 2003, it evaluates the range, frequency, and independence of activities. In Japan, the LSA was incorporated into the Elderly Status Assessment set (E-SAS), developed by the Japanese Physical Therapy Association during a nation project for elderly services between 2005 and 2007. Other measures identified in this review, such as TUG, the Falls Efficacy Scale (FES) (Tinetti, Richman, & Powell, 1990), and the Lubben Social Network Scale-6 (LSNS-6) (Lubben, 1988), were also incorporated into the E-SAS.

As indicated in the results, most outcome measures identified in this review were not specific to occupational therapy. The frequent use of tools for motor function, such as LSA, TUG, and FES, may reflect the team-based nature of rehabilitation outcomes. Several collaborative studies between physiotherapists and occupational therapists were identified. Only one tool was specifically designed for occupational therapy: the Classification and Assessment of Occupational Dysfunction (CAOD). Developed by Teraoka and Kyogoku, the CAOD demonstrated strong validity and reliability in studies with undergraduate participants (Teraoka & Kyogoku, 2015). It assesses occupational dysfunction, defined as negative experiences associated with daily activities (Teraoka & Kyogoku, 2014). The CAOD comprises 16 self-report items that capture four dimensions: occupational marginalisation, imbalance, deprivation, and alienation. Originally intended to prevent dysfunction among healthy individuals, it was later applied in a case report on a stroke survivor receiving home-visit rehabilitation to evaluate occupation-based practice ( Goto & Teraoka, 2019).

The findings addressing the primary question indicate that the frequent use of certain outcome measures is strongly influenced by public or governmental initiatives, suggesting that societal priorities may outweigh individual client needs. Furthermore, the limited availability of occupational therapy-specific tools implies that occupational therapists working in community settings for individuals with ABI in Japan tend to rely on general health outcome measures shared with other healthcare professionals. While the government-driven outcome measures are valuable for promoting collective and systematic improvement, their dominance may restrict the use of holistic assessments that are particularly important in occupational therapy. To strengthen professional identity and ensure that outcomes reflect clients’ needs, occupational therapy associations and service providers in Japan may needs to promote the complementary use of comprehensive assessment tools within clinical guidelines and routine documentation.

#### The concepts assessed by the extracted measurements

As shown in Appendix 6, most outcome measures extracted in this review assessed multiple concepts within a single tool, reflecting the multifaceted nature of ABI, which affects diverse functional domains. As illustrated in Fig. 3, Activity and Participation emerged as the most prominent ICF domain, with mobility and self-care being the most frequently assessed concepts in post-discharge occupational therapy for individuals with ABI in Japan.

In Japan, an ageing population combined with a shrinking caregiving workforce has made the shortage of carers a pressing societal concern (Ma & Yoshida, 2022). Consequently, promoting independence in both mobility and self-care has become a crucial rehabilitation goal. As ABI often impairs these areas, their prioritisation in community-based interventions is understandable.

However, it is equally important to address the needs of younger individuals with ABI living in the community. Although accurate national data are limited, a survey by Hachisuka et al. estimated that approximately 3,000 individuals aged 6–69 develop moderate cognitive dysfunctions due to brain injury each year in Japan (Hachisuka et al., 2011). The study also indicated that most of these individuals aim for social reintegration through rehabilitation. Yet, cognitive, behavioural, and communication difficulties often pose greater barriers to returning to home, school, or work than physical impairments. Outcome measures focusing solely on mobility and self-care may therefore fail to adequately capture rehabilitation progress of these individuals. Broader assessments that include household activities, employment, education, leisure, and interpersonal relationships may be required to guide more flexible and individualised community-based interventions.

Moreover, interventions focused exclusively on functional abilities may show diminishing effectiveness over time. Because ABI frequently results in permanent disabilities that cannot be fully resolved, rehabilitation should also assist individuals in achieving a satisfactory and meaningful life within a holistic framework. Tools that capture these broader and more subjective aspects of clients’ lives, particularly in the context of community-based rehabilitation, may therefore be necessary.

The review revealed a clear gap between administrative requirements and occupational therapy’s holistic approach. To address the needs of diverse community-dwelling individuals with ABI, occupational therapists could begin incorporating brief occupation-focused assessments alongside widely used functional indicators. In addition, future research should seek to develop and validate more comprehensive outcome measures suited to community rehabilitation in Japan. Studies examining how such tools can be integrated with existing government-mandated indicators would help ensure both clinical relevance and feasibility.

### Strengths and Limitations of this review

A key strength of this review lies in its inclusion of literature in both Japanese and English, an effort rarely seen in rehabilitation research. This represents an important step towards fostering international collaboration in the field.

As for limitations, this review included all detected articles, including grey literature, to capture both clinical and research perspectives. However, this approach may have introduced biases, particularly through the inclusion of conference booklets as grey literature. Because the databases searched did not comprehensively cover all rehabilitation-related conferences, the abstracts obtained were limited to specific events. To maintain feasibility, further manual searches were not undertaken. Therefore, the inclusion or exclusion of some grey literature may have influenced the findings.

## Conclusions

In rehabilitation, including occupational therapy for individuals with ABI in Japan, the FIM was identified as the most frequently used post-discharge tool, primarily assessing mobility and self-care. The frequent use of tools such as the FIM, FAI, LSA, and BI may reflect governmental priorities. These trends align with Japan’s ageing population, where independence in daily functioning is highly valued. However, ABI survivors, including younger individuals, often require broader support for community reintegration and personal goals. Incorporating additional assessment tools alongside those recommended by policy may help address these diverse needs. Occupational therapists, who work closely with clients in daily life, are well placed to promote the flexible use of person-centred outcome measurements that support meaningful participation and quality of life.

## Supplemental Information

10.7717/peerj.20765/supp-1 Supplemental Information 1 PRISMA checklist

10.7717/peerj.20765/supp-2 Supplemental Information 2 PCC framework for eligibility of studies

10.7717/peerj.20765/supp-3 Supplemental Information 3 Inclusion and Exclusion criteria

10.7717/peerj.20765/supp-4 Supplemental Information 4 Search strategy

10.7717/peerj.20765/supp-5 Supplemental Information 5 Source of Evidence (Japanese grey literature)

10.7717/peerj.20765/supp-6 Supplemental Information 6 Overview of the instruments identified across 44 included studies, including their names and the frequencies of use

10.7717/peerj.20765/supp-7 Supplemental Information 7 The 11 most frequently cited instruments (cited more than twice), categorised according to ICF domains

10.7717/peerj.20765/supp-8 Supplemental Information 8 1 Raw data full-text screening sheet A (English articles)

10.7717/peerj.20765/supp-9 Supplemental Information 9 2 Raw data full-text screening sheet B&C (Japanese articles)

## Additional Information and Declarations

### Data Availability

The following information was supplied regarding data availability: The raw data is available in the Supplemental Files.
