# Supplementary material for: Post-discharge outcome measurement tools in occupational therapy for people with acquired brain injury in Japan: a scoping review
Source: PeerJ. 2026 Mar 17;14:e20765. doi: 10.7717/peerj.20765 (PMC13003951; doi:10.7717/peerj.20765)
Supplement: Supplemental Information 2 [file peerj-14-20765-s002.docx]

| **Criteria** | **Determinants** |
| --- | --- |
| P (Population) | People with ABI |
| C (Concept) | Outcome measures  Occupational Therapy, Rehabilitation |
| C (Context) | Community, After discharge  Japan |
