# Supplementary material for: Post-discharge outcome measurement tools in occupational therapy for people with acquired brain injury in Japan: a scoping review
Source: PeerJ. 2026 Mar 17;14:e20765. doi: 10.7717/peerj.20765 (PMC13003951; doi:10.7717/peerj.20765)
Supplement: Supplemental Information 3 [file peerj-14-20765-s003.docx]

|  | **Inclusion criteria** | **Exclusion criteria** |
| --- | --- | --- |
| Study design | No restrictions | N/A |
| Country | Any locations in Japan | Outside Japan |
| Languages | No restrictions | N/A |
| Years | No restrictions | N/A |
| Participants | People with ABI who had rehabilitation in the community after discharge from hospitals | a) People without ABI  b) People who did not receive rehabilitation  c) Inpatients with ABI |
| Outcome measurement tools that are used | Outcome measurement tools related to rehabilitation, including occupational therapy | a) Tools that are not validated in Japan  b) Tools that are not focused on rehabilitation (i.e., medical treatment, pharmacy, nursing, dentistry, veterinary medicine)  c) Tools not focused on ABI (i.e., orthopaedic, cardiac, respiratory, infectious diseases).  d) Tools used to assess only specific functions (i.e., gait, limb movement, trunk stability, swallowing, language, cognitive functions including driving abilities)  e) Tool used only during hospitalisation (included only if also applied continuously after discharge) |
