# Supplementary material for: Post-discharge outcome measurement tools in occupational therapy for people with acquired brain injury in Japan: a scoping review
Source: PeerJ. 2026 Mar 17;14:e20765. doi: 10.7717/peerj.20765 (PMC13003951; doi:10.7717/peerj.20765)
Supplement: Supplemental Information 4 [file peerj-14-20765-s004.docx]

**Appendix**

| **Database** | **Search strategy** |
| --- | --- |
| MEDLINE Ultimate | S1. (outcome* OR "outcome* tool*" OR "outcome* assessment*" OR "outcome* evaluation*") AND (rehabilitation OR "occupational therapy" OR "occupational therapist*" OR ot) AND ("brain injur*" OR "acquired brain injury" OR "traumatic brain injury" OR abi OR tbi OR stroke OR "cerebrovascular accident" OR cva ) AND Japan AND (community OR after discharge)  S2. (outcome* OR "outcome* tool*" OR "outcome* assessment*" OR "outcome* evaluation*") AND ("Rehabilitation" OR "occupational therapy" OR "occupational therapist*" OR ot) AND ("brain injur*" OR "acquired brain injury" OR "traumatic brain injury" OR abi OR tbi OR stroke) AND Japan AND (community OR after discharge)  S3. (outcome* OR "outcome* tool*" OR "outcome* assessment*" OR "outcome* evaluation*") AND ("Rehabilitation" OR "occupational therapy" OR "occupational therapist*" OR ot OR health care) AND ("brain injury" OR "acquired brain injury" OR "traumatic brain injury" OR abi OR tbi OR stroke) AND Japan AND (community OR after discharge)  S4. (outcome* OR "outcome* tool*" OR "outcome* assessment*" OR "outcome* evaluation*") AND ("Rehabilitation" OR "occupational therapy" OR "occupational therapist*" OR ot OR health care) AND ("brain injury" OR "acquired brain injury" OR "traumatic brain injury" OR abi OR tbi OR stroke) AND Japan |
| Ichushi web  （医中誌web） | *English translations are provided following the original Japanese keywords.  #1 ("アウトカム評価(保健医療)"/TH or アウトカム/AL)  #2　 帰結/AL  #3　 帰結予測/AL  #4 (リハビリテーション/TH or リハビリテーション/AL)  #5 (作業療法/TH or 作業療法/AL)  #6 (脳卒中/TH or 脳卒中/AL)  #7 (脳損傷/TH or 脳損傷/AL)  #8 (脳血管障害/TH or 脳血管障害/AL)  #9 (日本/TH or 日本/AL)  #10 退院後/AL  #11 地域/AL  #12 #1 or #2 or #3  #13 #4 or #5  #14 #6 or #7 or #8  #15 #10 or #11  #16 #12 and #13 and #14 and #15  English translations  #1 ("outcome assessment"/TH or outcome/AL)  #2　 outcome/AL  #3　 outcome prediction/AL  #4 (rehabilitation/TH or rehabilitation/AL)  #5 (occupational therapy/TH or occupational therapy/AL)  #6 (stroke/TH or stroke/AL)  #7 (brain injury/TH or brain injury/AL)  #8 (cerebrovascular disease/TH or cerebrovascular disease/AL)  #9 (Japan/TH or Japan/AL)  #10 post-discharge/AL  #11 community/AL  #12 #1 or #2 or #3  #13 #4 or #5  #14 #6 or #7 or #8  #15 #10 or #11  #16 #12 and #13 and #14 and #15 |
