# Supplementary material for: Post-discharge outcome measurement tools in occupational therapy for people with acquired brain injury in Japan: a scoping review
Source: PeerJ. 2026 Mar 17;14:e20765. doi: 10.7717/peerj.20765 (PMC13003951; doi:10.7717/peerj.20765)
Supplement: Supplemental Information 5 [file peerj-14-20765-s005.docx]

| **Authors and Year** | **Name of instrument used** | **Outcome domain assessed** | **The number of items** | **Mode of Administration** | **Study design** | **Target population** | **Sample size** | **Age range and mean** | **Language** |
| --- | --- | --- | --- | --- | --- | --- | --- | --- | --- |
| Hirose & Suzuki, 2020 | FIM | ADL, motor function, cognitive function, communication | 18 subscales (eating, grooming, bathing, dressing upper body, dressing, lower body, toileting, bladder management, bowel management, transfers-bed/chair/wheelchair, transfers-toilet, transfers-bath/shower, walk/wheelchair, stairs, comprehension, expression, social interaction, problem solving, memory) | observation | retrospective observational study | home visit rehabilitation clients with CVA discharged from the convalescent rehabilitation ward or the community-based care ward | 15 | 74.3±6.9 | Japanese |
| Mada et al., 2010 | FIM | ADL, motor function, cognitive function, communication | 18 subscales (eating, grooming, bathing, dressing upper body, dressing, lower body, toileting, bladder management, bowel management, transfers-bed/chair/wheelchair, transfers-toilet, transfers-bath/shower, walk/wheelchair, stairs, comprehension, expression, social interaction, problem solving, memory) | observation | case report | a day care client with CVA | 1 | 68 | Japanese |
|  | SDS | mood | 20 questions related to signs of depression | self-report |  |  |  |  |  |
|  | QUIK-R | QOL | 55 questions (20 for physical function, 10 for emotional adaptation, 10 for social relationship, and 10 for goal of life) *there are 5 additional questions related to characteristics | self-report |  |  |  |  |  |
| Toyama et al., 2009 | FIM | ADL, motor function, cognitive function, communication | 18 subscales (eating, grooming, bathing, dressing upper body, dressing, lower body, toileting, bladder management, bowel management, transfers-bed/chair/wheelchair, transfers-toilet, transfers-bath/shower, walk/wheelchair, stairs, comprehension, expression, social interaction, problem solving, memory) | observation | survey report (symposium abstract) | home visit rehabilitation clients with CVA discharged from the convalescent rehabilitation ward | 25 | not mentioned | Japanese |
| Karasuno et al., 2004 | FIM | ADL, motor function, cognitive function, communication | 18 subscales (eating, grooming, bathing, dressing upper body, dressing, lower body, toileting, bladder management, bowel management, transfers-bed/chair/wheelchair, transfers-toilet, transfers-bath/shower, walk/wheelchair, stairs, comprehension, expression, social interaction, problem solving, memory) | observation | retrospective observational study | People with CVA living at home | 86 | not mentioned | Japanese |
| Toyama, 2010 | FIM | ADL, motor function, cognitive function, communication | 18 subscales (eating, grooming, bathing, dressing upper body, dressing, lower body, toileting, bladder management, bowel management, transfers-bed/chair/wheelchair, transfers-toilet, transfers-bath/shower, walk/wheelchair, stairs, comprehension, expression, social interaction, problem solving, memory) | observation | survey report (symposium abstract) | home visit rehabilitation with CVA | 25 | not mentioned | Japanese |
| Sato et al., 2008 | FIM | ADL, motor function, cognitive function, communication | 18 subscales (eating, grooming, bathing, dressing upper body, dressing, lower body, toileting, bladder management, bowel management, transfers-bed/chair/wheelchair, transfers-toilet, transfers-bath/shower, walk/wheelchair, stairs, comprehension, expression, social interaction, problem solving, memory) | observation or interview | retrospective cohort study | stroke outpatients discharged from the acute ward to their home | 32 | 71.1±8.5 | Japanese |
| Toyonaga, 2010 | mRS | the degree of disability or dependence (for people who suffered from a stroke or other neurological diseases) | a single scale | observation | case control study | people with CVA discharged from a hospital | 109 (return to work＝48, not return to work＝ 61) | not mentioned | Japanese |
|  | BI | ADL, motor function | 10 subscales (feeding, bathing, grooming, dressing, bowels, bladder, toilet use, transfers, mobility, stairs) | observation |  |  |  |  |  |
|  | MMSE | cognitive function | 11 questions to assess cognitive impairment (orientation, attention, short-term memory, language, visuospatial abilities, understanding instructions) | interview |  |  |  |  |  |
| Watanabe et al., 2005 | PCRS | self-awareness for ADL, IADL, cognitive function, communication, and emotion | 30 questions to assess recognition for their own ability to perform and manage various daily activities and situations | self-report and report from their family | controlled before after study | clients with severe TBI received a group rehabilitation programme for social skill training | 6 | not mentioned | Japanese |
| Hashimoto et al., 2005 | FIM/FAM | ADL, motor function, cognitive function, communication, IADL | 30 subscales (eating, grooming, bathing, dressing upper body, dressing, lower body, toileting, swallowing, bladder management, bowel management, transfers-bed/chair/wheelchair, transfers-toilet, transfers-bath/shower, transfers-car, walk/wheelchair, stairs, community access, comprehension, expression, reading, writing, speech intelligibility, social interaction, emotional status, adjustment to limitations, employability, problem solving, memory, orientation, attention, safety judgement) | observation | controlled before after study | clients with ABI received a day care rehabilitation programme | 5 | not mentioned | Japanese |
|  | GAF | mental health (social, psychological and occupational function) | a single scale |  |  |  |  |  |  |
| Tokizato et al., 2005 | SF-36 | health related QOL | 36 questions to assess 8 areas (physical functioning, role physical, bodily pain, general health, vitality, social functioning, role emotional, mental health) | self-report | retrospective observational study | stroke survivors using a day care or a home visit rehabilitation | 60 | 70.2 (±9.4) | Japanese |
| Kasai et al., 2005 | FIM | ADL, motor function, cognitive function, communication | 18 subscales (eating, grooming, bathing, dressing upper body, dressing, lower body, toileting, bladder management, bowel management, transfers-bed/chair/wheelchair, transfers-toilet, transfers-bath/shower, walk/wheelchair, stairs, comprehension, expression, social interaction, problem solving, memory) | staff | survey study | stroke outpatients after hospitalised rehabilitation | 32 | 62 | Japanese |
|  | TMIG-IC | IADL | 13 items (transportation, shopping, preparing for meals, payments, managing finances, managing official documents, reading newspaper, reading books or magazines, being interested in articles or TV related to health, visiting friends, listening to others to help, visiting hospitals to see others, talking to young people) |  |  |  |  |  |  |
| Seki, 2005 | BI | ADL, motor function | 10 subscales (feeding, bathing, grooming, dressing, bowels, bladder, toilet use, transfers, mobility, stairs) | interview | survey study | stroke outpatients | 82 | 70.9 | Japanese |
| Sawa et al., 2020 | FIM | ADL, motor function, cognitive function, communication | 18 subscales (eating, grooming, bathing, dressing upper body, dressing, lower body, toileting, bladder management, bowel management, transfers-bed/chair/wheelchair, transfers-toilet, transfers-bath/shower, walk/wheelchair, stairs, comprehension, expression, social interaction, problem solving, memory) | observation | retrospective observational study | stroke survivors with 15 years after onset | 28 | 71.4 (±6.2) | Japanese |
|  | FAI | IADL | 15 subscales including 36 items (preparing main meals, washing up after meals, washing clothes, light housework, heavy housework, local shopping, social occasions, walking outside for >15minutes, actively pursuing hobby, driving a car/going on a bus, travel outing/car ride, gardening, household maintenance, reading books, gainful work) |  |  |  |  |  |  |
| Suzuki et al., 2020 | FIM | ADL, motor function, cognitive function, communication | 18 subscales (eating, grooming, bathing, dressing upper body, dressing, lower body, toileting, bladder management, bowel management, transfers-bed/chair/wheelchair, transfers-toilet, transfers-bath/shower, walk/wheelchair, stairs, comprehension, expression, social interaction, problem solving, memory) | observation | case report | a stroke survivor used a day care rehabilitation service | 1 | 75 | Japanese |
| Yokoi et al., 2015 | BI | ADL, motor function | 10 subscales (feeding, bathing, grooming, dressing, bowels, bladder, toilet use, transfers, mobility, stairs) | observation | survey study | young stroke outpatients of a rehabilitation centre more than a year after onset | 67 | range 18-39 | Japanese |
| Oikawa et al., 2016 | LSA | a range, frequency and independence level of activities | 15 questions regarding range, frequency and independence level in rooms of home beside the room for sleeping, an area outside home, places in neighbourhood, places outside neighbourhood, places outside a town | self-report | cross-sectional study | outpatients or day care clients with CVA | 115 | 61.7 (±11.7) | Japanese |
| Sonoyama et al., 2016 | FIM | ADL, motor function, cognitive function, communication | 18 subscales (eating, grooming, bathing, dressing upper body, dressing, lower body, toileting, bladder management, bowel management, transfers-bed/chair/wheelchair, transfers-toilet, transfers-bath/shower, walk/wheelchair, stairs, comprehension, expression, social interaction, problem solving, memory) | observation | retrospective observational study | home visit rehabilitation clients with CVA | 21 | 73.3 (±7.6) | Japanese |
| Tozima & Hashimoto, 2016 | BI | ADL, motor function | 10 subscales (feeding, bathing, grooming, dressing, bowels, bladder, toilet use, transfers, mobility, stairs) | observation and interview | controlled before after study | stroke outpatients discharged from the acute ward to their home | 60 | not mentioned | Japanese |
|  | Lawton IADL | IADL | 8 subscales (ability to use telephone, shopping, food preparation, housekeeping, laundry, mode of transportation, responsibility for own medication, ability to handle finances) |  |  |  |  |  |  |
|  | BBS/FBS | motor function | 14 items (sitting to standing, standing unsupported, sitting unsupported, standing to sitting, transfers, standing with eyes closed, standing with feet together, reaching forward with outstretched arm, retrieving object from floor, turning to look behind, turning 360 degrees, placing alternate foot on stool, standing with one foot in front, standing on one foot) | observation |  |  |  |  |  |
|  | SDMT | cognitive function | no item (the achievement percentage for the task) |  |  |  |  |  |  |
| Sazika et al., 2015 | SF-36 | health related QOL | 8 subscales (physical functioning, role physical, bodily pain, general health, vitality, social functioning, role emotional, mental health) | self-report | qualitative study | people with mild TBI more than a year after onset discharged from an acute ward | 31 | 34.7 | Japanese |
|  | PCRS | self-awareness for ADL, IADL, cognitive function, communication and emotion | 30 questions to assess clients' recognition for their own ability to perform and manage various daily activities and situations | self-report and report from others |  |  |  |  |  |
| Ito et al., 2021 | FAI | IADL | 15 subscales including 36 items (preparing main meals, washing up after meals, washing clothes, light housework, heavy housework, local shopping, social occasions, walking outside for >15minutes, actively pursuing hobby, driving a car/going on a bus, travel outing/car ride, gardening, household maintenance, reading books, gainful work) | observation | cross-sectional study | outpatients with brain tumour | 24 | not mentioned | Japanese |
|  | EORTC QLQ-C30 | health related QOL | 15 questions (global QOL, physical function, emotion, fatigue, nausea/vomiting, pain, breathing, insomnia, anorexia, constipation) | self-report |  |  |  |  |  |
|  | HADS | mood | 14 questions related to signs of depression (7 items for depression and 7 items for anxiety) | self-report |  |  |  |  |  |
