# Supplementary material for: Post-discharge outcome measurement tools in occupational therapy for people with acquired brain injury in Japan: a scoping review
Source: PeerJ. 2026 Mar 17;14:e20765. doi: 10.7717/peerj.20765 (PMC13003951; doi:10.7717/peerj.20765)
Supplement: Supplemental Information 6 [file peerj-14-20765-s006.docx]

FIM: Functional Independence Measure / LSA: Life Space Assessment / FAI: Frenchay Activities Index / BI: Barthel Index / SF-36: Mos Short Form-36 Health Survey / TUG: Time Up and Go Test /BBS, FBS: Berg Balance Scale, Functional Balance Scale / mRS: modified Rankin Scale / MMSE: Mini-Mental State Examination / PCRS: Patient Competence Rating Scale / FAM: Functional Assessment Measure / GDS-15: Geriatric Depression Scale-15 / FES: Falls Efficacy Scale / LSNS-6: Lubben Social Network Scale-6 / CAOD: Classification and Assessment of Occupational Dysfunction / 10MWT: 10 Meter Walking Test / 6MWT: 6 / Minute Walking Test / CS-30: 30 second chair stand test / POMS: Profile of Mood States / SDS: Self-rating Depression Scale / QUIK-R: Self Completed Questionnaire for Quality of Life Revised / GAF: Global Assessment of Functioning / TMIG-IC: Tokyo metropolitan institute of gerontology index of competence / Lawton IADL: Lawton Instrumental Activities of Daily Living Scale / SDMT: Symbol Digit Modality Test / EORTC QLQ-C30: European Organization for Research of Cancer Quality of Life Questionnaire / HDRS: Hamilton / Depression Rating Scale / SEPA: Self-Efficacy of Physical Activities / PSQI: Pittsburgh Sleep Quality Index / CIQ: Community Integration Questionnaire / HADS: Hospital Anxiety and Depression Scale

| **The name of instruments** | **The number of studies** |
| --- | --- |
| FIM | 23 |
| FAI | 7 |
| LSA | 6 |
| BI | 6 |
| SF-36 | 3 |
| TUG | 2 |
| BBS/FBS | 2 |
| mRS | 2 |
| PCRS | 2 |
| FIM/FAM | 2 |
| GDS15 | 2 |
| MMSE | 1 |
| FES | 1 |
| LSNS-6 | 1 |
| CAOD Scale | 1 |
| Flow FIM | 1 |
| 10MWT | 1 |
| 6MWT | 1 |
| CS-30 | 1 |
| POMS | 1 |
| SDS | 1 |
| QUIK-R | 1 |
| GAF | 1 |
| TMIG-IC | 1 |
| Lawton IADL | 1 |
| SDMT | 1 |
| EORTC QLQ-C30 | 1 |
| HADS | 1 |
| the SEPA scale | 1 |
| PSQI | 1 |
| CIQ | 1 |
| HDRS | 1 |
|  | 78 |
