# Supplementary material for: Post-discharge outcome measurement tools in occupational therapy for people with acquired brain injury in Japan: a scoping review
Source: PeerJ. 2026 Mar 17;14:e20765. doi: 10.7717/peerj.20765 (PMC13003951; doi:10.7717/peerj.20765)
Supplement: Supplemental Information 7 [file peerj-14-20765-s007.docx]

|  |  | **FIM** | **LSA** | **FAI** | **BI** | **SF-36** | **TUG** | **BBS/**  **FBS** | **mRS** | **PCRS** | **FIM/**  **FAM** | **GDS-15** |
| --- | --- | --- | --- | --- | --- | --- | --- | --- | --- | --- | --- | --- |
| Body function | mental function |  |  |  |  | ● |  |  |  | ● | ● | ● |
|  | sensory functions and pain |  |  |  |  | ● |  |  |  |  |  |  |
|  | functions of the digestive, metabolic and endocrine system |  |  |  |  |  |  |  |  |  | ● |  |
| Activity and Participation | learning and applying knowledge | ● |  |  |  |  |  |  |  | ● | ● |  |
|  | communication | ● |  |  |  |  |  |  |  | ● | ● |  |
|  | mobility | ● | ● | ● | ● | ● | ● | ● | ● | ● | ● |  |
|  | self-care | ● |  |  | ● | ● |  |  | ● | ● | ● |  |
|  | interpersonal interactions and relationship | ● |  |  |  | ● |  |  |  | ● | ● |  |
|  | domestic life |  |  | ● |  |  |  |  |  | ● |  |  |
|  | major life areas |  |  | ● |  |  |  |  |  |  | ● |  |
|  | community, social and civic life |  |  | ● |  |  |  |  |  |  |  |  |
